# Feasibility and Initial Efficacy Evaluation of a Community-Based Cognitive-Behavioral Lifestyle Intervention to Prevent Excessive Weight Gain During Pregnancy in Latina Women

**DOI:** 10.1007/s10995-015-1698-x

**Published:** 2015-02-10

**Authors:** Sabina B. Gesell, Jeffrey A. Katula, Carmen Strickland, Mara Z. Vitolins

**Affiliations:** 1 Division of Public Health Sciences, Department of Social Sciences and Health Policy, Medical Center Boulevard, Wake Forest School of Medicine, Winston-Salem, NC 27157 USA; 2 The Maya Angelou Center for Health Equity, Wake Forest School of Medicine, Winston-Salem, NC USA; 3 Department of Health and Exercise Science, Wake Forest University, Winston-Salem, NC USA; 4 Family and Community Medicine, Wake Forest School of Medicine, Winston-Salem, NC USA; 5 Division of Public Health Sciences, Department of Epidemiology and Prevention, Wake Forest School of Medicine, Winston-Salem, NC USA

**Keywords:** Gestational weight gain, Maternal health, Pregnancy, Obesity, Hispanic Americans

## Abstract

About 48 % of US women gain more weight during pregnancy than recommended by the Institute of Medicine (IOM). Excessive gestational weight gain is a major risk factor for obesity in both women and offspring over their lifetimes, and should be avoided. This study was designed to test the feasibility and initial efficacy of a prenatal behavioral intervention in a sample of low-income, predominantly Latina women. The intervention was delivered in groups of 8–10 women in a community recreation center, and structured to reduce the proportion of women who gained weight in excess of IOM guidelines. Recruitment targets were met in 3 months: 135 pregnant women (>10 and <28 weeks) were randomly assigned to receive a 12-week intervention (n = 68) or usual care (n = 67). Retention rate was 81 %. On average, women attended 4 of 12 group sessions, and each session had 4 of the 8–10 assigned participants in attendance. Initial efficacy analyses were based on 87 women. Compared to usual care, fewer normal-weight women in the intervention exceeded IOM recommendations (47.1 % usual care vs. 6.7 % intervention; absolute difference 40.4 %; *p* = .036). Recommendations for recruitment, retention, and delivery are discussed. A community-based cognitive-behavioral lifestyle intervention during pregnancy was feasible in a hard-to-reach, high-risk population of low-income Latina women, and showed efficacy in preventing excessive gestational weight gain. Due to frequently changing work schedules, strategies are needed to either increase attendance at group sessions (e.g., within a group prenatal care format) or to build core skills necessary for behavior change through other modalities.

**Electronic supplementary material:**

The online version of this article (doi:10.1007/s10995-015-1698-x) contains supplementary material, which is available to authorized users.

## Introduction

The Centers for Disease control and prevention (CDC) estimates that 48 % of United States (US) women gain more weight during pregnancy than recommended by the Institute of Medicine (IOM) [1]. The IOM recommends that underweight women (BMI < 18.5) gain 28–40 lb, normal-weight women (BMI = 18.5–24.9) gain 25–35 lb, overweight women (BMI = 25.0–29.9) gain 15–25 lb, and obese women (BMI > 30) gain 11–20 lb during pregnancy. Excessive gestational weight gain (GWG) is a major risk factor for postpartum weight retention, which contributes to new and persistent maternal obesity [2–5] and perpetuates a cycle of maternal-infant health complications with each subsequent pregnancy. Excessive GWG is independently associated with neonatal adiposity [6] and greater body mass index (BMI) in childhood, adolescence, and early adulthood [7–10], although the intra-uterine mechanisms involved are still unclear [11].

In its 2009 report, the IOM focused on the need for effective, sustainable GWG interventions [2]. To date, such interventions are typically clinic-based and have had mixed success [12]. The most recent meta-analysis of interventions on GWG found a 1.42 kg reduction (95 % confidence interval 0.95 to 1.89 kg, *p* < 0.001) in GWG interventions versus controls [13], with no significant difference between intervention and control groups in adherence to IOM recommendations. Correspondingly, there are scant evidence-based recommendations for clinical practice in antenatal care [14], and there remains an urgent need for effective, sustainable interventions focused on healthy GWG.

Hispanic and African–American women are at increased risk of entering into pregnancy overweight [15] and gaining additional weight during their childbearing years, both during and following pregnancies [4, 16, 17]. Hispanic women also have increased fertility rates [18]. However, GWG interventions have focused on non-Hispanic White women, a situation that the IOM found especially noteworthy [19]. Variations in intervention dose, timing and method of delivery, quality of study designs [20], and effects within subgroups of women (based on BMI, age, ethnicity, parity, underlying medical conditions, and socioeconomic status) [21] complicate evaluation of previous GWG interventions.

This study was designed to evaluate feasibility and initial efficacy of a 12-week GWG intervention among low-income minority women. We hypothesized that women who received the intervention would be less likely to exceed IOM pregnancy weight gain recommendations than a usual-care control group. Because understanding how successful interventions achieve outcomes is an important—yet generally underreported—aspect of designing more effective programs [22], we also provide programmatic details about the program.

## Methods

This project (called Madre Sana, Bebé Sano/Healthy Mother, Healthy Baby) was conducted in collaboration with Nashville Parks and Recreation. The study was approved by an Institutional Review Board and registered at ClinicalTrials.gov. Recruitment occurred between January and April 2011. Participants provided written consent in their language of choice (Spanish or English).

### Recruitment

Our goal was to recruit 100 women. We developed referral systems with community and hospital clinics with Spanish-speaking obstetricians. Women were eligible if they were >10 and<28 weeks pregnant, ≥16 years old, in prenatal care, Spanish- or English-speaking, expecting to remain in Middle Tennessee for their entire pregnancy, and willing to sign a release form for medical record abstraction. There were no exclusion criteria based on the number of prior pregnancies or other medical conditions. We also developed a referral system for women who were interested in participating but not in prenatal care, which connected them to a medical home and made them study-eligible within several weeks. Most participants were enrolled in clinic waiting rooms. Others contacted us after being referred through social service providers (e.g., WIC offices, Catholic Charities).

### Retention

To support session attendance, we used strategies proven effective in our previous studies [23, 24], including (1) starting intervention sessions within 2 weeks of enrollment; (2) scheduling group sessions at convenient times and encouraging make-up sessions; (3) offering transportation and childcare; (4) providing food during sessions; (5) inexpensive incentives for attendees (e.g., diapers, toys); and (6) raffle prizes ($100 strollers) at the last session, with odds favoring those participants who attended the most sessions. Participants also received a nominal gift at each measurement visit (e.g., $12 baby blankets and Mexican *rebozos*). Retention strategies included weekly telephone calls and text messages from the interventionists, and following a standardized protocol regarding missed sessions or unreachable participants.

### Randomization

Because degree of overweight/obesity may influence the outcomes of interest [19], randomization was stratified based on pre-pregnancy BMI category, using measured height and self-reported pre-pregnancy weight. We used block randomization in groups of 2 to ensure that each arm remained balanced for pre-pregnancy BMI. The randomization sequence was computer-generated by a data manager who did not meet potential participants during recruitment. After randomization, the data manager documented the group assignment and informed study coordinator of the assignment; a bilingual research assistant then called the participant to give them their group assignment. To ensure adequate allocation concealment, the randomization sequence list was kept centrally with the data manager.

### Setting

Intervention sessions were held at a community recreation center operated by the Parks and Recreation Department in Nashville’s highest minority concentration area. The recruitment clinics also pulled from this catchment area. This choice offered a built environment supporting the desired behavioral changes, (1) reinforced the principle of using one’s built environment to promote and potentially sustain healthy lifestyles, and (2) facilitated dissemination of the intervention (if effective) to other community centers.

### Control Condition

All participants received the control intervention; the intervention group also received the healthy lifestyle intervention. The control intervention was an infant injury prevention intervention using the best-practice based “A New Beginning” curriculum, delivered in three 30-min home visits (at baseline, week 6, week 12) [25]. A treatment fidelity plan ensured that GWG and related behaviors (physical activity, nutrition, sleep hygiene) were not discussed to avoid contamination of experimental conditions. The control group interventionists were hospital interpreters with nuanced understanding of the multiple Hispanic cultures of the participants.

### Intervention

Women in the intervention arm attended 12 weekly 90-min group sessions (8–10 women and one facilitator). Two bilingual, trained healthcare providers delivered the intervention.

### Theoretical Framework and Conceptual Model

#### Social Learning Theory (SLT)

SLT [26–29] focuses on learning within a social context, hypothesizing that all behavior we display socially is learned primarily by observing and imitating others’ behaviors and associated rewards and punishments. In a social learning environment, participants explore new ways of thinking, practice health skills, and acquire positive attitudes about health via modeling with one another [30, 31].

#### The Core Competency Model

Skills-based interventions, grounded in SLT, seek to promote acquisition of core competencies. The core competencies in our program were behavior change strategies (i.e., decision making, goal setting, self-monitoring, rewarding successful behavior, self-efficacy enhancement, problem solving and relapse prevention) focused on nutrition, exercise, sleep hygiene, coping with stress and anxiety, communication, money and time management, social skills, and assertiveness. Best practices in instructional design for adults were used to promote active learning, retention, and transfer (practical application in new contexts) of knowledge, skills, and attitudes (KSAs). KSAs enable learners to demonstrate behaviors in group sessions that facilitate effective and confident performance in real-world situations.

### Curriculum Development Process

The curriculum was created by a professional curriculum developer and modeled after the effective Botvin LifeSkills^®^ Training program, which focuses on preventing alcohol and tobacco use via development of personal and social competencies [32]. We (1) defined the core competencies needed to modify physical activity, nutrition, and sleep behaviors; (2) operationalized a training model to define KSA statements for each competency, (3) created a framework and drafted each lesson; and (4) modified and finalized each lesson after several practice sessions.

We convened three focus groups of Latina women who were either pregnant or postpartum (evaluating their GWG in hindsight) and a community advisory board. The board included health and social service providers who serve low-income families, and highly connected community members. The focus groups refined the intervention and curriculum content; discussed barriers and facilitators of the study (e.g., meaningful incentives, spousal acceptance of home visits; influence of culture on prenatal health practices); and examined assumptions about cultural relevance, language, meaning, and comprehension [33]. The final intervention was a manualized cognitive-behavioral curriculum incorporating this feedback.

### Curriculum Content

The curriculum is detailed in Appendix S1. The delivery sequence constitutes the learning scaffold, which asks the learner to acquire foundational KSAs in the lesson (competency) area. Each successive lesson asks the learner to apply what was previously learned to the new area of inquiry. Mastery in the competencies results from continuous, and increasingly challenging, reinforcement of KSAs.

We included major components of previous interventions to prevent excessive GWG in our curriculum (e.g., setting weight goals, tracking weight with the participant, offering frequent feedback and encouragement, physical activity programming, nutritional counseling). Our intervention also included components critical to obesity prevention but new to GWG interventions (e.g., sleep hygiene, shopping/cooking skills, money management skills, highlighting offerings in public recreation centers, systematic practice of behavior change skills). Throughout, we highlighted family values, traditions, and experiences (comparing and contrasting experiences in their country of origin and in the US), to connect desired behaviors with cultural norms and values and highlight cultural strengths to support behavior change. For example, the first session started with conversation about (1) cultural influences on decision-making around food, exercise and sleep during pregnancy; (2) how some of these influences support health and others do not; and (3) how to use a step-by-step model to decide the best course of action for each woman.

Each session included: (1) health education; (2) learning and practicing a core competency necessary to successfully manage weight, including self-management skills (e.g., impulse control, problem solving, time and money management, coping with stress and anxiety); social skills (building positive support among family and friends for healthy living); obesity resistance skills (awareness of cultural influences, providing prevention-related education); (3) a group exercise class; (4) a group cooking class; (5) building a supportive social network; and (6) measuring and tracking weight. Appendix S2 describes the tools used.

### Fidelity Plan

A treatment fidelity plan was developed based on suggestions from the Treatment Fidelity Workgroup of the NIH Behavior Change Consortium [34]. The plan included interventionist training and supervision; identifying essential treatment components for verification; sampling to ensure treatment consistency; and collecting fidelity measures to monitor and enhance reliability and validity of the intervention.

### Data Collection

Feasibility and fidelity metrics were collected throughout the trial as process measures. Survey data were collected by bilingual, trained study staff via interview in participants’ homes at baseline, Week 6, and Week 12. Medical charts were abstracted at the end of the trial.

### Measures

#### Feasibility and Fidelity

Feasibility was measured by ability to: recruit and retain participants who were willing to be randomized and complete the 3 interviews during home visits; and obtain both obstetric and pediatric medical charts to abstract outcomes. Fidelity was measured by the length, number, frequency of sessions, delivery of full educational content and practice activities in the pre-specified order, and participant attendance at the study protocol specified group sessions.

#### Pre-Pregnancy BMI

BMI (weight [kg]/height [m^2^]) [19] was used for block randomization. BMI was calculated using calculators from the CDC [35, 36] using measured height and self-reported pre-pregnancy weight at enrollment. The validity of self-reported pre-pregnancy weight is high [37–40]. Phelan and colleagues reported a correlation of *r* = 0.95 between participant self-reported and physician-measured weights (*p* = 0.0001) with a mean discrepancy of 0.5 ± 3.0 kg, and no significant differences between healthy weight and overweight/obese women (*p* = 0.64) [39]. Height was measured by trained research staff with a portable stadiometer (Charder HM-200P Portstad) at baseline.

#### Adherence to IOM Recommendations

Weight at last prenatal care visit was abstracted from medical records after delivery. GWG was computed using standard methods [41] and the 2009 IOM recommendations. We classified GWG as 0 (below weight gain recommendations), 1 (within weight gain recommendations), or 2 (above weight gain recommendations) based on pre-pregnancy BMI. The primary outcome was the proportion of women who exceeded IOM recommendations. These data were collected for effect size estimation and precise powering of a subsequent full trial.

### Statistical Analysis

Feasibility metrics are reported as proportions. We used Chi square tests to test for group differences on GWG category. We examined the efficacy of the intervention stratified by pre-pregnancy BMI category, because pre-pregnancy BMI is the single best predictor of GWG, and GWG targets vary by pre-pregnancy BMI [42–44]. ANOVA main effects and interaction effects were used to examine differences between groups on the secondary outcomes, stratified by pre-pregnancy BMI.

## Results

### Feasibility and Fidelity

**Fig. 1:**
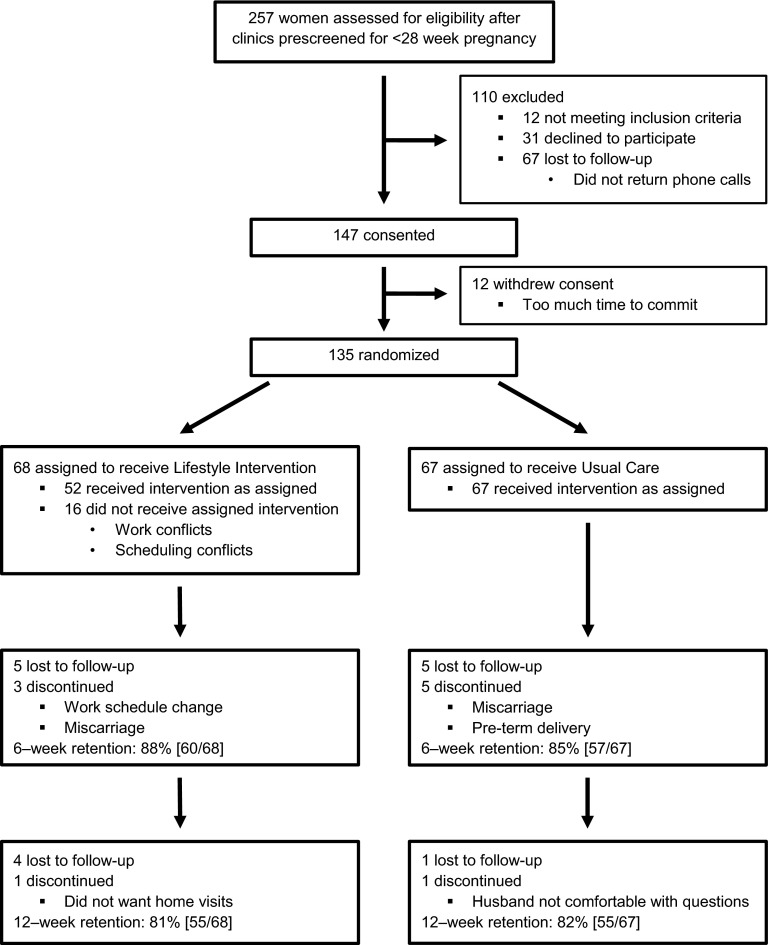
CONSORT diagram: Flow of participant recruitment and retention

We assessed 257 prescreened women for eligibility through in-person interviews; 135 (53 %) were randomized into the study within 3 months. Rate of enrollment was higher when study team members spoke with potential participants face-to-face (62 %) than by telephone (37 %). Figure 1 shows the flow of participants through the trial. Overall, 110 completed all three interviews (81 % retention rate). We did not observe differential attrition between study arms.

Adherence to intervention sessions ranged from 0 % (0/12) to 100 % (12/12). On average, women attended 4.14 (SD = 3.85) of the 12 sessions (Median = 3, Mode = 0, Table 1). On average, each session had 4.17 (SD = 1.67) attendees. Participants’ first sessions were offered within 2 weeks of enrollment, with several makeup sessions offered. At enrollment, participants were asked for preferred time and days to attend sessions; these were offered mornings, afternoons, and evenings on weekdays as requested.

**Table 1:** Attendance at protocol-specified group sessions by intervention arm participants (N = 68)

Number of sessions attended	n	%	Cumulative %
0	16	23.5	23.5
1	9	13.2	36.8
2	4	5.9	42.6
3	7	10.3	52.9
4	6	8.8	61.8
5	1	1.5	63.2
6	5	7.4	70.6
7	4	5.9	76.5
8	4	5.9	82.4
9	1	1.5	83.8
10	6	8.8	92.6
11	3	4.4	97.1
12	2	2.9	100.0
Total	68	100.0	

We obtained obstetric charts for 87/135 (64 %) participants. Of these participants, 74 also had infant birth weight abstracted from pediatric charts. We found no significant differences in study arm assignment, pre-pregnancy BMI category, race, language preference, or education between participants for whom we did or did not have medical records (Table 2). Participant demographics are shown in Table 3.

**Table 2:** Baseline characteristics of women with and without the variables of interest (gestational weight gain, birth weight, gestational age) via chart abstraction (N = 130)

	Had all three follow-up variables measured through chart abstraction				Chi square *p* value
	Yes		No		
	n	%	n	%	
Total	74	56.9	56	43.1	
Condition					
Intervention	36	58.5	29	41.5	0.7232
Control	38	55.4	27	44.6	
Baseline weight					
Normal	29	58.0	21	42.0	0.8207
Overweight	24	53.3	21	46.7	
Obese	21	60.0	14	40.0	
Race					
Latina	58	61.1	37	38.9	0.1211
African–American	7	36.8	12	63.2	
Other	8	66.7	4	33.3	
Language preference					
English	16	45.7	19	54.3	0.2921
Spanish	53	60.9	34	39.1	
English AND Spanish	5	62.5	3	37.5	
Education					
Less than high school degree	45	60.0	30	40.0	0.8919
High school or equivalent	18	64.3	10	35.7	
More than high school	11	57.9	8	42.1

**Table 3:** Baseline characteristics of women for whom obstetric medical charts were obtained, by randomization condition (N = 87)

Characteristics	Control (n = 43) n (%)	Intervention (n = 44) n (%)	Chi square *p* value
Race			
Hispanic	32 (37)	37 (43)	0.306
Non-Hispanic White	4 (5)	1 (1)	
African–American	5 (6)	3 (3)	
Asian	0 (0)	0 (0)	
Other	1 (1)	3 (3)	
WIC recipient	17 (20)	19 (22)	0.730
Country of origin			
US	12 (14)	6 (7)	0.181
Ecuador	1 (1)	0 (0)	
El Salvador	3 (3)	3 (3)	
Guatemala	2 (2)	0 (0)	
Honduras	6 (7)	6 (7)	
Mexico	15 (17)	26 (30)	
Puerto Rico	2 (2)	0 (0)	
Other	2 (2)	3 (3)	
Smoked cigarettes while pregnant	2 (2)	2 (2)	0.381
Marital status			
Currently married and living together	31 (36)	37 (43)	0.015
Never married	6 (7)	0 (0)	
Geographically separated	1 (1)	5 (6)	
Separated/single	5 (6)	2 (2)	
Self-reported food insecurity			
Sometimes run out of food before able to buy more	20 (23)	29 (33)	0.068
Cannot afford to eat healthy	12 (14)	10 (11)	0.578
Need help obtaining food	13 (15)	15 (17)	0.700
	Control (n = 43) Mean (SD)	Intervention (n = 44) Mean (SD)	
Age	25.86 (5.982)	27.55 (5.817)	0.186
Prior deliveries	1.19 (1.484)	1.52 (1.285)	0.261

Two interventionists delivered the active healthy lifestyle intervention, and 3 delivered the control injury prevention intervention. All were trained and certified; all educational content and planned activities fully occurred in sessions, verified by study team observers, and that intervention content was never discussed with control group participants.

### Intervention Effect

To help establish consistency in the reporting of effect sizes from GWG interventions, we present the GWG outcome as both a categorical variable (adherence to IOM guidelines) and as a continuous variable (reduction in total weight gained). This feasibility study was not powered to detect either treatment effects or adverse events.

#### Adherence to IOM Guidelines

Fewer women exceeded IOM weight gain recommendations in the intervention group the control group, although this difference was not statistically significant (Table 4). Based on pre-pregnancy BMI category, the intervention effect was statistically significant for normal weight women, but not for overweight women or obese women.

**Table 4:** Gestational weight gain relative to IOM recommendations for women for whom obstetric medical charts were obtained (N = 87)

Pre-pregnancy BMI category		Control (n = 43) n (%)	Intervention (n = 44) n (%)	Chi square	*p* value
Normal	Under IOM rec	6 (35.3)	8 (53.3)	6.631	0.036
	Within IOM rec	3 (17.6)	6 (40.0)		
	Over IOM rec	8 (47.1)	1 (6.7)		
Overweight	Under IOM rec	6 (40.0)	3 (21.4)	2.969	0.227
	Within IOM rec	3 (20.0)	7 (50.0)		
	Over IOM rec	6 (40.0)	4 (28.6)		
Obese	Under IOM rec	1 (9.1)	4 (26.7)	1.669	0.434
	Within IOM rec	5 (45.5)	4 (26.7)		
	Over IOM rec	5 (45.5)	7 (46.7)		
All	Under IOM rec	13 (30.2)	15 (34.1)	2.998	0.223
	Within IOM rec	11 (25.6)	17 (38.6)		
	Over IOM rec	19 (44.2)	12 (27.3)		

#### GWG as Continuous Variable

Mean GWG was 22.41 lb (SD = 15.56, Min −24.88, Max 52.94) for control participants and 19.50 lb (SD = 12.27, Min −7.44, Max 53.40) for intervention participants. A *t* test for equality of means yielded a non-significant difference in total weight gain between groups (*t* = − 0.894, *p* = 0.374). For comparison with published studies, this analysis included participants whose weight was recorded in their medical record within 2 weeks of delivery [45] (control n = 36, intervention n = 38).

#### Examination of Adverse Effect on Birth Outcomes

The intervention did not affect birth weight (*p* = 0.9641) or gestational age at birth (*p* = 0.4653), and interaction terms with pre-pregnancy BMI also were not statistically significant (*p* = 0.3668, *p* = 0.3979, respectively). We observed no differences between groups for birth weight or gestational age, among women who gained under, within, or over IOM recommendations.

## Discussion

This pilot study in Latina women was shown to be feasible. Recruitment and retention rates were very high. Recruiting Latinos into a research study is challenging and in part, why they have been underrepresented in research [46–49]. Our recruitment strategy relied on referrals from a trusted source, such as a Spanish-speaking healthcare provider or community leader. Potential participants were contacted initially either in person at their provider’s office or by phone as a referral. At the point of contact a brief summary of the program was presented. When the initial contact was by phone, an in-person meeting was arranged as quickly as possible to continue the process. All participants received a small incentive (key chain) for listening to the brief summary. For participants interested in enrolling, a more detailed explanation was given. Twenty to 30 min was devoted to reviewing the consent form, both to fully inform participants and to build trust to support retention. These processes resulted in high recruitment and retention rates. Further our data show recruiting potential participants was more successful when the initial contact was face-to-face versus by telephone.

Our retention strategy relied on building relationships and trust with our study participants, which required maintaining continuity of study team members [23], being accessible at any time during the day and on weekends, and making participants feel important throughout the study. While interest in study enrollment was high, attendance at group sessions was mixed, even though we went to great lengths to overcome barriers by providing transportation, car seats, on-site childcare, and make-up sessions at varying times including evening hours (refer to Retention section). A significant barrier to session attendance was participants having no control over their own or their husband’s often-changing work schedules, particularly because typically they were given only 1 day’s advance notice. All this suggests it will be important to find new ways to increase attendance at group sessions or to build core skills necessary for behavior change through other modalities (e.g., online, mobile phone, DVD, home visits) when engaging this vulnerable population in research.

One approach that would reduce participant burden—and thus perhaps increase session attendance and intervention “dose”—is to connect GWG education and skills-building support to prenatal care visits. An innovative alternative to individual counseling interventions is to weave GWG modules into group prenatal care sessions. We have reported that group prenatal care reduced the risk of excessive GWG to 54 % of what it would have been in the standard model of individual prenatal care (NNT = 5) in low-income minority women [50]. The CenteringPregnancy group prenatal care curriculum includes education around prenatal nutrition and exercise (among other wellness topics); the group format is intended to provide social support and facilitate collective problem-solving around barriers to behavior change. Future research should examine the extent to which focused GWG education and skills-building within group prenatal care might further reduce excessive weight gain during pregnancy.

Retention, as defined by proportion of participants who completed data collection at each wave, was high. Rather than requiring participants to attend data collection sessions in the community center, we conducted home visits for data collection for all three waves. This was based on feedback from our community advisory board regarding acceptability of home visits. Even participants who did not attend intervention sessions participated in data collection in their home. Although more resource-intensive, home visits for data collection reduced missing data. The findings also demonstrate initial efficacy to support powering for future studies.

Although this feasibility study was not powered to detect differences between groups, and results must be interpreted with caution, the intervention reduced the proportion of normal weight women who gained weight in excess of IOM recommendations, which few studies to date have achieved [20, 51]. The intervention’s initial effectiveness in normal weight and overweight women are promising trends for further investigation. Also warranting further attention in a larger trial is the proportion of women who gain below IOM recommendations. In addition to the reduced lifetime risk of obesity in mother and child, many other significant adverse health outcomes, along with their associated healthcare costs, could also be averted by an effective and practical GWG intervention.

### Strengths and Limitations

This study successfully recruited Latina women, who are significantly under-represented in similar studies to date. This study used a curriculum which had received significant input and refinement from our focal population. One limitation was low session attendance, which highlights the need to anticipate attendance barriers and develop creative solutions when engaging this population in research. Another limitation was missing data on the primary outcome for a full-scale trial, which highlights the challenges of medical record abstraction in trials. In a recent review [52] the proportion of medical records successfully obtained ranged from 50 to 85 % for single [53, 54] and multi-site studies [55]. Continuously monitoring whether participants switch prenatal care providers would reduce challenges in obtaining outcomes from medical records after delivery. The observed loss to follow-up may be due to the study’s design, which did not require participants to attend at least two baseline study visits before randomization, as is typically done in efficacy trials. The generalizability of results may be limited to Latina women. However, information from this feasibility trial will inform a future, adequately powered randomized lifestyle intervention trial for pregnant Latinas, who are at greater risk for negative sequelae years after their pregnancies (as are their offspring). The preliminary efficacy of this work coupled with the need for effective, practical GWG interventions, especially for low-income and minority women, highlight the need for a larger trial focused on this high-risk population.

## Conclusion

A community-based cognitive-behavioral lifestyle intervention during pregnancy is feasible in a hard-to-reach, high-risk subpopulation of low-income minority women and may prevent excessive GWG. The greatest challenge is ensuring sufficient dose through increased attendance at group sessions, or developing behavior change skills through other modalities.

## Electronic supplementary material

Below is the link to the electronic supplementary material.

Supplementary material 1 (DOCX 22 kb)

Supplementary material 2 (DOCX 540 kb)
