# Supplementary material for: Feasibility and Initial Efficacy Evaluation of a Community-Based Cognitive-Behavioral Lifestyle Intervention to Prevent Excessive Weight Gain During Pregnancy in Latina Women
Source: Matern Child Health J. 2015 Feb 10;19(8):1842–52. doi: 10.1007/s10995-015-1698-x (PMC4500842; doi:10.1007/s10995-015-1698-x)
Supplement: Supplementary file 1 — Supplementary material 1 (DOCX 22 kb) [file 10995_2015_1698_MOESM1_ESM.docx]

**Appendix S1**

| **Madre Sana, Bebé Sano Curriculum Scope and Sequence** | |
| --- | --- |
| 1. **Cultural Influences and Decision Making** | 1. Provide an overview of the sessions and objectives of the program |
|  | 1. Introduce participants to the exercise facility and engage in group walking exercise |
|  | 1. Identify how culture and folklore influences decision making about food, exercise, and sleep |
|  | 1. Apply the decision making model to a personal decision related to food, exercise, and sleep |
|  | 1. Prepare a healthy snack |
|  | 1. State what was learned during the session and identify opportunities to practice decision making in the upcoming week |
|  | 1. Re-enforce identity as a social network for improving health |
| 1. **Food, Exercise, and Sleep: Ingredients of a Healthy Pregnancy** | 1. Review basic clinical guidelines about of food, exercise, and sleep during pregnancy |
|  | 1. Engage in 15 minutes of group exercise |
|  | 1. Develop a definition to distinguish healthy from unhealthy behaviors and beliefs about food, exercise, and sleep |
|  | 1. Describe the connection between food, exercise, and sleep in a healthy pregnancy |
|  | 1. Review the skill of self-monitoring to identify targets and actions for changing unhealthy food, exercise, and sleep behaviors |
|  | 1. Learn the steps for goal setting |
|  | 1. Apply the goal setting skill to change a personal, unhealthy practices related to food, exercise, and sleep |
|  | 1. Prepare a healthy snack |
|  | 1. State what was learned during the session and identify opportunities to practice the goal setting skill to change a weight-related health behavior during the program |
|  | 1. Re-enforce identity as a social network for improving health |
| 1. **Food** | 1. State the risks and examine the benefits of weight gain management during pregnancy |
|  | 1. Engage in 15 minutes of group exercise |
|  | 1. Re-assess the extent to which previously identified folklore about weight and weight gain are accurate |
|  | 1. Identify simple rules for decreasing the amount of sugar, fat, and highly processed foods in the diet |
|  | 1. Apply a step by step goal setting method for decreasing sugar, fat, and highly processed food in the diet by implementing the food rules |
|  | 1. Explore tips for how to achieve goals |
|  | 1. Prepare a healthy snack |
|  | 1. State what was learned during the session and identify opportunities to practice the goal setting skill to change a personal weight-related health behavior during the program |
|  | 1. Re-enforce identity as a social network for improving health |
| 1. **Movement and Exercise** | 1. Acquire knowledge, skills, and attitudes that promote engagement in exercise and physical activity |
|  | 1. Engage in 15 minutes of group exercise |
|  | 1. Distinguish the difference between exercise and activity |
|  | 1. Identify and refute myths and folklore about exercise and activity during pregnancy |
|  | 1. Describe the benefits of exercise and activity |
|  | 1. Develop a list of exercises and activities that they can do alone or with others. |
|  | 1. Identify tips for overcoming the barriers to exercise and making it a part of their day |
|  | 1. Apply the step by step goal setting procedure to increase exercise and activity |
|  | 1. Prepare a healthy snack |
|  | 1. State what was learned during the session and identify opportunities to practice the goal setting skill to change a personal health behavior related to exercise during the program |
|  | 1. Re-enforce identity as a social network for improving health |
| 1. **Sleep and Rest** | 1. Acquire knowledge, skills, and attitudes that promote good sleep and rest |
|  | 1. Engage in 15 minutes of group exercise |
|  | 1. Understand the effects of pregnancy on sleep |
|  | 1. Identify barriers to good sleep and tips for success |
|  | 1. Examine 10 strategies for improving the quality and quantity of sleep and rest |
|  | 1. Apply the step by step goal setting procedure to implement three strategies to improve sleep and rest practices |
|  | 1. Prepare a healthy snack |
|  | 1. State what was learned during the session and identify opportunities to practice the goal setting skill to change a personal health behavior related to sleep |
|  | 1. Re-enforce identity as a social network for improving health |
| 1. **Making the Most of Time and Money** | 1. Identify personal values that are influenced by time and money |
|  | 1. Engage in 15 minutes of group exercise |
|  | 1. Examine how time and money are resources that help achieve personal values |
|  | 1. Develop a list of simple organizational skills |
|  | 1. Apply organizational skills to manage time and money to achieve goals and reflect personal values |
|  | 1. Consider how organizational skills can be applied to prioritizing sleep and exercise |
|  | 1. Apply organizational skills to plan Session 12, The Community Pot Luck |
|  | 1. Prepare a healthy snack |
|  | 1. State what was learned during the session and identify opportunities to practice organizational skills for managing time and money during the week |
|  | 1. Re-enforce identity as a social network for improving health |
| 1. **Managing Stress and Anxiety** | - 1. Engage in 15 minutes of group exercise |
|  | - 1. Examine effects of food, exercise, and sleep on both triggering and reducing stress and anxiety during pregnancy |
|  | - 1. Identify stress triggers that are both personally unique as well as universal during pregnancy |
|  | - 1. Identify the physical and other emotional symptoms of stress and anxiety |
|  | - 1. Distinguish between healthy and unhealthy reactions to stress triggers |
|  | - 1. Review and practice the steps for three stress management techniques: Deep Breathing, Centering, and the Double Standard |
|  | - 1. State the benefits of healthy management of stress and anxiety |
|  | - 1. Apply organizational skills from Session 7 to plan Session 12, The Community Pot Luck |
|  | - 1. Prepare a healthy snack |
|  | - 1. State what was learned during the session and identify opportunities to practice organizational skills for managing time and money during the week |
|  | - 1. Re-enforce identity as a social network for improving health |
| 1. **Communication** | 1. Engage in 15 minutes of group exercise |
|  | 1. Generate a list of the situations that arise during pregnancy where communication is important to health |
|  | 1. Identify the reasons for misunderstandings in communications with others |
|  | 1. Review and apply verbal and non-verbal communication skills that reduce and prevent misunderstandings and get needs met |
|  | 1. Review and practice four techniques that reduce and prevent misunderstandings and get needs met |
|  | 1. Predict the benefits to self and others of healthy communication practices |
|  | 1. Apply organizational skills from Session 7 and communication skills from Session 8 to plan Session 12, The Community Pot Luck |
|  | 1. Prepare a healthy snack |
|  | 1. State what was learned during the session and identify opportunities to practice communication skills |
|  | 1. Re-enforce identity as a social network for improving health |
| 1. **Building a Support Network** | 1. Engaged in 15 minutes of group exercise |
|  | 1. Identify personal sources and types of support for prenatal health |
|  | 1. Identify gaps in personal support networks |
|  | 1. Describe the benefits and attributes of supportive relationships |
|  | 1. Review the components of healthy support networks |
|  | 1. Identify qualities in self and others that support the development of healthy friendships and social networks |
|  | 1. Reviewed skills that build new and strengthen existing circles of support |
|  | 1. Review and practice skills for inviting people to do things and responding to invitations from others |
|  | 1. Apply social skills to plan Session 12, The Community Pot Luck |
|  | 1. Prepare a healthy snack |
|  | 1. State what was learned during the session and identify opportunities to practice skills that build healthy friendships and support networks |
|  | 1. Re-enforce identity as a social network for improving health |
| 1. **Understanding Assertiveness** | 1. Engage in 15 minutes of group exercise |
|  | 1. Identify situations around food, exercise, sleep and medical care where it is difficult to be assertive |
|  | 1. Understand the role of influence and persuasion |
|  | 1. Define and distinguish passive, aggressive and assertive verbal and non-verbal behaviors |
|  | 1. Review and apply assertive verbal and non-verbal behaviors to a current situation related to diet, exercise, sleep, or medical care |
|  | 1. Apply skills acquired in Sessions 6 - 10 to plan Session 12, The Community Pot Luck |
|  | 1. Prepare a healthy snack |
|  | 1. State what was learned during the session and identify opportunities to practice verbal and non-verbal assertiveness skills during the week |
|  | 1. Re-enforce identity as a social network for improving health |
| 1. **Be Assertive** | 1. Engage in 15 minutes of group exercise |
|  | 1. Review the steps for three assertiveness techniques: Say No, Solve the Problem, and Start with “I” |
|  | 1. Practice each technique using a current challenge to being assertive |
|  | 1. Reflect on the appropriate situations for using each technique |
|  | 1. Apply skills acquired in Sessions 6 - 11 to plan Session 12, The Community Pot Luck |
|  | 1. Prepare a healthy snack |
|  | 1. State what was learned during the session and identify opportunities to practice the three assertiveness techniques skills during the week |
|  | 1. Re-enforce identity as a social network for improving health |
| 1. **Celebrating Our Success** | 1. Participate in a community celebration of successes achieved during the program |
|  | 1. Share accomplishments and lessons learned with others in the participants’ family and other social networks |
|  | 1. Encourage ongoing communication and engagement with one another |
