# Supplementary material for: Feasibility and Initial Efficacy Evaluation of a Community-Based Cognitive-Behavioral Lifestyle Intervention to Prevent Excessive Weight Gain During Pregnancy in Latina Women
Source: Matern Child Health J. 2015 Feb 10;19(8):1842–52. doi: 10.1007/s10995-015-1698-x (PMC4500842; doi:10.1007/s10995-015-1698-x)
Supplement: Supplementary file 2 — Supplementary material 2 (DOCX 540 kb) [file 10995_2015_1698_MOESM2_ESM.docx]

**Appendix S2**

**Instructional Approach**

In developing the framework for each lesson, we emphasized best practices in instructional design and adult learning.[[31](#_ENREF_31),[32](#_ENREF_32)] **Each lesson had four types of learning tasks that supported cognitive-behavioral change and developed efficiacy in the use of new skills.** The types of tasks were:

1. Anchor Task: Invites the participants to construct meaning based on what they already know or believe about the topic.
2. Add Task: Invites the participants to consider new information and concepts and presents them with procedures and techniques for new skills.
3. Apply Task: Asks the learners to use what they have learned to create new understandings and practice new behaviors.
4. Away Task: Invites learners to practice what they have learned in their real world environment.

These tasks build on and are linked to one another throughout the program, creating continuous reinforcement of key, pro-health knowledge, skills, and attitudes.

**Just as important as what is taught, are the teaching strategies that are used to deliver the program.** Lecture based instruction has been found to be ineffective in developing efficacy for health behavior change. The Madre Sana program utilized four teaching strategies that are particularly effective where social learning and cognitive-behavioral change are the desired results. These four skills and their application were:

1. Facilitation: Develop or expand ideas and concepts and examine biases and errors in thinking.
2. Coaching: Organize peer-to-peer practice of new thinking and behaviors.
3. Behavioral Rehearsal: Connect thoughts to actions through rehearsal of skills using real world contexts.
4. Feedback: Assessment of understanding through reflection on performance by self, peers and facilitator.

Coaching checklists used to support interventionists in applying these teaching strategies are provided below.

## Facilitation

## Facilitation was the teaching skill we used when we wanted to discover existing knowledge, introduce new ideas and concepts, and correct errors and biases in thinking about a topic. Facilitators were instructed to go through the following steps:

- - State purpose of discussion
  - Clarify goals
  - Stimulate and steer discussion by asking open-ended questions
    - - - Non-intimidating questions
        - Allow sufficient "wait time"
  - Correct misinformation
  - Keep order
  - Give positive feedback
  - Maintain comfort and monitor self-disclosure
  - Closure/Summary

## Coaching

## Coaching was the teaching skill we used when we wanted learners to develop new skills and behaviors. Facilitators were instructed to go through the following steps:

## ⬜ Define goal

⬜ Give explicit, step-by-step instructions of behavior

⬜ Demonstrate behavior correctly (teacher or student)

⬜ Review step-by-step instruction

⬜ Organize practice (in small groups, pairs, or individually)

⬜ Elicit and give feedback

⬜ Reinforce effective behavior

⬜ Closure: Facilitator has final word - bring it back to goal

**Behavioral Rehearsal**

Behavioral Rehearsal is the teaching skill we used to organize, conduct, and process peer-to-peer practice of pro-health behaviors.

**Directing a behavioral rehearsal.**

⬜ State purpose and define goal.

⬜ Recruit volunteer(s).

⬜ Describe skill and scenario or situation, and give volunteers specific instructions.

⬜ Model skill, or direct volunteers to model skill.

⬜ Practice: Set an explicit time limit and have *all* students practice the skills.

⬜ Coach students by giving and eliciting suggestions and specific directions, and repeat practice, if necessary.

**Processing a behavioral rehearsal.**

⬜ Ask the class for feedback using the Four Open Questions:

- Observe: *“What happened?” “What did you notice?”*
- Analyze: *“Why do things like this often happen?” “What worked? What didn’t?”*
- Predict: *“What could happen next?”*
- Suggest: *“What are some other choices?” “What are your suggestions?”*

⬜ Synthesize learning: Identify skill components and assess their effectiveness.

⬜ Closure: Summarize and bring it back to goal.

**Tips for successful behavioral rehearsals.**

- Keep it simple: short, structured and skill-specific.
  - Focus on skill practice.
  - Keep directions and scenarios simple; avoid “hot button” issues.
  - Focus on the skill being taught, not “solving” a problem or scenario.

⬜ Model positive, pro-social behaviors, not problem behaviors.

⬜ Ask students to be themselves, not someone else. *(This increases the chances of a skill being generalized to a real-life situation.)*

⬜ Apply Social Learning Theory through peer observation, feedback, and practice.

⬜ Spend twice as much time processing the skills as you did practicing them.

**Feedback**

Feedback is an assessment method. This feedback model is a step-by-step procedure for organizing and conducting feedback.
